# Fecal Microbiota Transplantation (FMT) in the Management of Ulcerative Colitis: A Comprehensive Systematic Review and Meta-Analysis of Randomized Controlled Trials (RCTs)

**DOI:** 10.7759/cureus.111804

**Published:** 2026-06-30

**Authors:** Amal Ahad, Sandeep Kumar, Hugh Kolomar, Jada Williams, Abrar I Abdallah, Amirali Sadeghzadegan, Dana Yateem, Punam Kharel, Dristy Chowdhury, Fahad Alnajar, Muqaddas Ali

**Affiliations:** 1 Department of Endocrinology, Pinderfields Hospital, Wakefield, GBR; 2 Department of Medicine, Calderdale and Huddersfield NHS Foundation Trust, Huddersfield, GBR; 3 Department of Medicine, Niš Clinical Centre, Nis, SRB; 4 Department of Medicine, St. George's University, Brooklyn, USA; 5 Department of Surgery, Sulaiman Alhabib Hospital, Jeddah, SAU; 6 Department of Medicine, Marmara University School of Medicine, Istanbul, TUR; 7 Department of Trauma and Orthopaedics, The Shrewsbury and Telford Hospital NHS Trust, Shrewsbury, GBR; 8 Department of Respiratory Medicine, Medway NHS Foundation Trust, Gillingham, GBR; 9 Department of Hematology and Oncology, Medway NHS Foundation Trust, Gillingham, GBR; 10 Department of Medical Education, Mohammed Bin Rashid University of Medicine and Health Sciences (MBRU), Dubai, ARE; 11 Department of Medicine, Medipol University, Beykoz, TUR

**Keywords:** clinical remission, endoscopic remission, fecal microbiota transplantation, meta-analysis, ulcerative colitis

## Abstract

Ulcerative colitis (UC) is a chronic inflammatory disorder of the colon with increasing global prevalence, particularly in newly industrialized countries. Fecal microbiota transplantation (FMT) has worked as an effective therapeutic strategy aimed at restoring immune homeostasis and gut microbial balance. However, a lack of standardized protocols and comprehensive safety data necessitates further evaluation.

This meta-analysis aims to comprehensively analyze the efficacy of FMT in inducing clinical remission in UC, incorporating the latest randomized controlled trials (RCTs). A systematic review and meta-analysis of RCTs investigating FMT in UC were conducted. The primary outcomes were clinical and endoscopic remission, while adverse events (AEs) were assessed as secondary outcomes. Pooled risk ratios (RRs) with 95% confidence intervals (CIs) were calculated, and heterogeneity was analyzed using the I^2^ statistic and Cochran's Q test.

Overall, FMT demonstrated a significant benefit in inducing clinical remission compared with placebo (RR 1.55; 95% CI 1.22-1.96; p = 0.0003). For endoscopic remission, FMT showed a significant overall effect (RR 1.68; 95% CI 1.15-2.46; p = 0.007). The incidence of AEs was comparable between the FMT and control groups (RR 0.88; 95% CI 0.77-1.00; p = 0.06).

This meta-analysis gives strong confirmation for the efficacy of FMT in inducing both clinical and endoscopic remission in UC patients, with a favorable safety profile. Multi-donor FMT and oral capsule administration appear to be particularly promising. Future research should focus on standardizing protocols, elucidating mechanisms of action, and conducting larger, long-term trials to optimize FMT for UC.

## Introduction and background

Ulcerative colitis (UC) is a chronic inflammatory disease and an idiopathic condition, with current understanding indicating involvement of the colonic mucosa. UC usually begins at the rectum and spreads contiguously to the entire colon but may extend beyond the rectum. There are also patients who are diagnosed with proctitis or left-sided colitis who may also have a patch of inflammation in the cecum [1,2].

The global burden of inflammatory bowel disease (IBD) is significant but has notable regional differences. Siew et al.'s study reported the highest values of UC prevalence in Europe, with Norway having a prevalence of 505 per 100,000 cases and North America with 286 per 100,000 cases. In North America, Oceania, and several countries in Europe, the overall prevalence of IBD is more than 0.3%. A majority of studies (83.3%) have shown a stable, declining, or geographically restricted increase in IBD prevalence in North America and Europe, unlike the newly industrialized countries in the 1990s and present (particularly the newly industrialized countries of the global South, including Brazil as a representative of South America) [3]. Current guidelines for the management of UC of mild to moderate severity focus on the usage of 5-aminosalicylates.

For patients with moderate or severe 5-aminosalicylic acid therapy treatment failure, treatment usually consists of corticosteroids, followed by tapering to a steroid-sparing therapy, which may include thiopurines, anti-tumor necrosis factor (anti-TNF) agents, or receptor antagonists [2]. Although these medications are effective, they come with unpleasant side effects. For example, sulfasalazine, a common medication for patients with UC, can cause male infertility [4]. Corticosteroids can cause osteoporosis or anemia [5].

The causes of UC are complex. They include genetic causes, dysbiosis of microbes, and environmental factors [6]. In the colon and distal small intestine, the microbiome, which consists of thousands of billions of bacteria, including anaerobes, facultative anaerobes, and aerobes, is a complex ecosystem [4]. They create biofilms on the surfaces of the epithelial membranes of the gut, share and shape the membranes of the epithelial lines, and play a role in the permeability, immune function, and extras [7]. It is crucial to maintain a delicate equilibrium within the microbial ecosystem of the intestine to prevent the growth of potentially harmful organisms and minimize health risks. Intestinal microbiota dysbiosis can impair the immune response of the intestine, promote the growth of harmful organisms, and lead to the invasion of the mucosal layer, thus exacerbating the disease course of UC [7].

Fecal microbiota transplantation (FMT) is an innovative therapy involving the transplantation of gut microbiota from healthy individuals into a diseased gut. FMT aims to restore bacterial symbiosis in the recipient’s intestinal tract by increasing the diversity of their gut microbiota. In doing this, a new assembly of gut microbiota is established, the immune response becomes less dysregulated, and intestinal mucosal permeability is improved [8]. In addition, FMT protects the gut mucosa by activating a humoral immune response that results in the formation of immunoglobulin (Ig) A, IgG, and IgM. FMT also reduces the release of some pathogenic inflammatory cytokines. Also, FMT is associated with decreased intestinal pH, which is beneficial for the adhesion of good (beneficial) bacteria in the intestinal tract and also helps in the competitive inhibition of pathogenic bacteria (pathogens), which in turn helps to restore a healthy gut microbiota by enhancing the population of good bacteria and increasing diversity of the microbiota [7,8].

Despite the promising potential and numerous systematic reviews on FMT for UC, significant challenges remain. The literature currently lacks a standardized protocol for microbiota administration [9], and safety considerations exhibit inconsistencies across studies [10]. Moreover, critical factors such as optimal donor characteristics, patient selection criteria, and the ideal timing of FMT remain undefined [11]. Crucially, the specific active components within the donor microbiome responsible for the therapeutic effects of FMT have yet to be identified [12].

The present analysis intends to close these gaps by evaluating the most recent randomized controlled trials (RCTs) on the efficacy of FMT in achieving remission in UC, evaluating the safety of FMT in patients with UC, and determining the effectiveness of FMT with respect to the selection of the donor, the method of FMT administration, and the number of FMT procedures. Formulating a standard protocol for FMT for UC continues to be an important area of research. While systematic reviews published between 2023 and 2025 have confirmed the broad therapeutic utility of FMT, they lack granular subgroup breakdowns regarding specialized delivery pathways (such as lyophilized oral capsules) and optimized donor stratification models. Following the PICO framework, this study evaluates adult patients with active UC (Population) undergoing FMT (Intervention) compared to placebo controls (Comparison) to measure pooled rates of clinical/endoscopic remission and adverse event (AE) frequencies (Outcomes).

## Review

Methods

**Figure 1:**
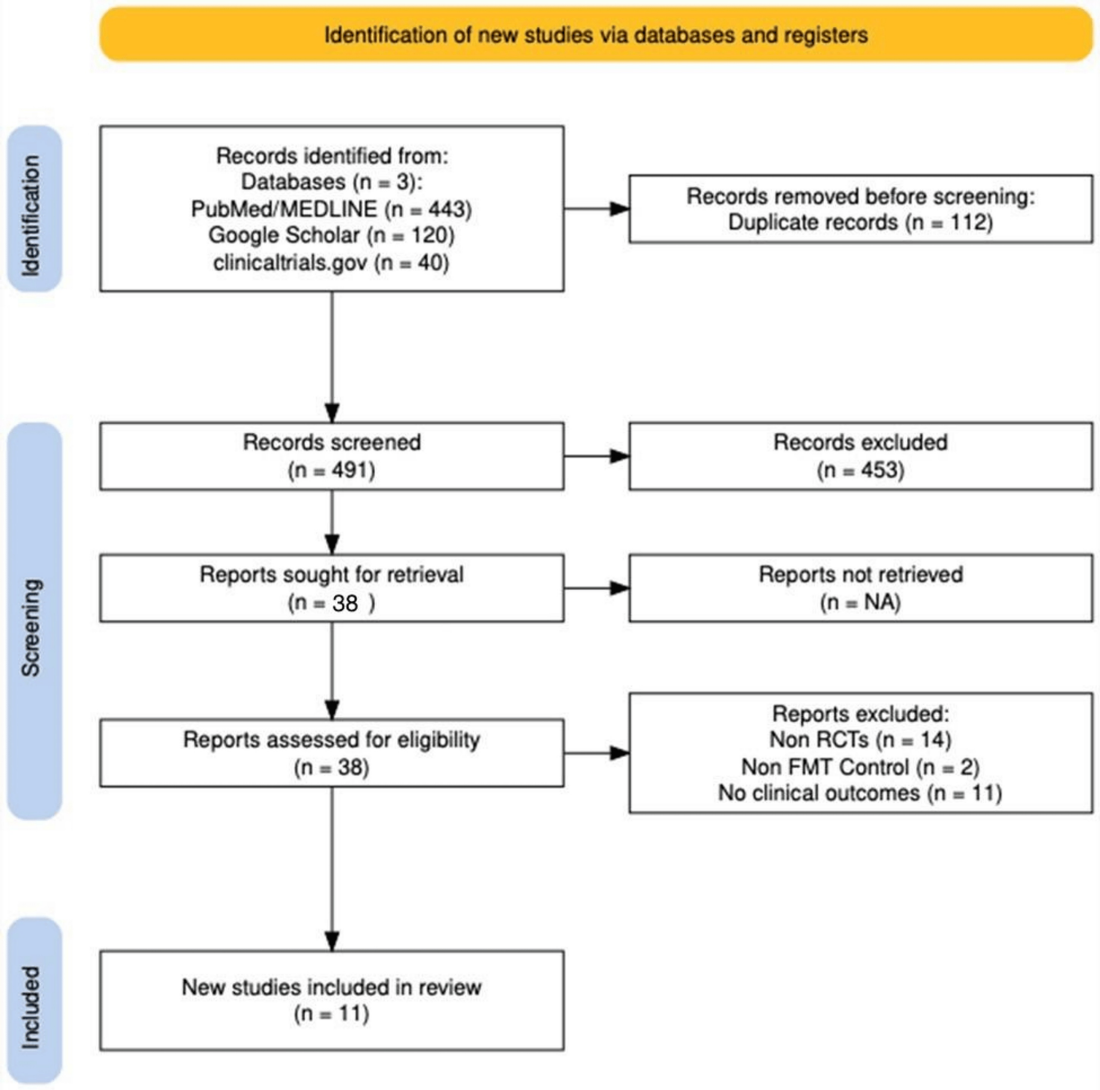
PRISMA Flow Chart of the Study Selection Process RCT: randomized controlled trial; FMT: fecal microbiota transplantation

In accordance with the PRISMA guidelines [13], an extensive search of the literature was conducted in these given databases: PubMed/MEDLINE, Google Scholar, and clinicaltrials.gov. Appropriate Boolean operators were utilized to combine the search terms: “Fecal microbiota,” “ulcerative colitis,” “gut microbiota,” “FMT,” and “GMT.” The literature cited in selected studies was used to confirm the comprehensiveness of the search. The strategy of search used for the study is provided in Table 1. RCTs with clinical outcomes in adult patients who had received FMT or placebo were selected. Table 2 provides the detailed inclusion and exclusion criteria. Titles and abstracts were screened by SK and FA, two independent reviewers. MA and AIA, two independent reviewers, conducted the full-text screening. Conflicts in either screening were addressed and resolved by a third reviewer, MA. Figure 1 is a PRISMA flow chart of the selection process of the study, as established by [14].

**Table 1:** Search Strategy

Database	Search terms/strings
PubMed/MEDLINE	("Fecal Microbiota Transplantation"[MeSH Terms] OR "Fecal microbiota"[tiab] OR "FMT"[tiab] OR "Gut Microbiota"[tiab] OR "GMT"[tiab]) AND ("Colitis, Ulcerative"[MeSH Terms] OR "Ulcerative colitis"[tiab] OR "UC"[tiab])
Google Scholar	allintitle: ("fecal microbiota transplantation" OR "FMT" OR "gut microbiota") AND ("ulcerative colitis" OR "UC")
clinicaltrials.gov	Condition or disease: Ulcerative colitis OR UC Other terms: Fecal microbiota transplantation OR FMT OR gut microbiota

**Table 2:** Inclusion & Exclusion Criteria FMT: fecal microbiota transplantation

Category	Criteria
Inclusion criteria	Randomized controlled trials (RCTs); adult patients; FMT or placebo intervention
Exclusion criteria	Non-RCTs, observational studies, reviews, editorials, commentaries, conference abstracts; pediatric patients; interventions other than FMT; no placebo/control group; insufficient or unextractable data

Two reviewers, JW and AA, evaluated the quality of methodology of the studies. In addition, risk of bias (RoB) for included studies was investigated through the Cochrane RoB2 tool. Statistical analysis has been performed with RevMan 5.4.1 (The Cochrane Collaboration, London, UK). Primary endpoints included remission (clinical and endoscopic), and secondary endpoints included AEs. Descriptive statistics were used for comparisons. Risk ratio (RR) was estimated with binary random effects to account for variability among included studies. Heterogeneity was evaluated with I^2^ statistics. The manuscript has been prepared and reported according to AMSTAR (A MeaSurement Tool to Assess systematic Reviews) [15] and TITAN (Transparency In The reporting of Artificial iNtelligence) [16] guidelines.

Results

Baseline Characteristics

A total of 11 [17-27] RCTs with 467 patients (236 FMT group; 231 placebo group) were included in our review. By continent of publication, the majority were from Asia (four); the remaining studies were from North America (three), Oceania (two), and Europe (two). In terms of delivery method, FMT was administered through colonoscopy (six studies), enema (four), and oral capsules (one). The baseline characteristics of the included studies are presented. Table 3 reports the baseline characteristics of the included studies. Table 4 reports patient-level baseline characteristics (eight studies).

**Table 3:** Baseline Characteristics

Study	Continent	Route of administration	Donor type	Evaluation duration
Lahtinen et al., 2023 [11]	Europe	Colonoscopy	Multiple	Week 48
Paramsothy et al., 2017 [17]	Oceania	Colonoscopy + enema	Multiple	Week 8
Moayyedi et al., 2015 [18]	North America	Enema	Single	Week 7
Kedia et al., 2022 [19]	Asia	Colonoscopy	Multiple	Week 8
Sarbagili Shabat et al., 2022 [20]	Asia	Colonoscopy + enema	Single	Week 8
Haifer et al., 2022 [21]	Oceania	Oral capsules	Single	Week 8
Crothers et al., 2021 [22]	North America	Colonoscopy + oral capsules	Multiple	Week 12
Březina et al., 2021 [23]	Europe	Enema	Single	Week 12
Pai et al., 2021 [24]	North America	Enema	Multiple	Week 30
Fang et al., 2021 [25]	Asia	Colonoscopy	Single	Week 8
Schierová et al., 2020 [26]	Europe	Enema	Single	Week 12
Sood et al., 2019 [27]	Asia	Colonoscopy	Single	Week 48

**Table 4:** Patient-Level Baseline Characteristics 5-ASA: 5-aminosalicylic acid (mesalamine); FMT: fecal microbiota transplantation; FMT-AID: fecal microbiota transplantation plus anti-inflammatory diet protocol; IQR: interquartile range; optimized SMT: optimized standard medical therapy; SCCAI: Simple Clinical Colitis Activity Index; FT: fecal transplantation; IBDQ: Inflammatory Bowel Disease Questionnaire

Study	Cohort/arm	Sample size (N)	Mean age (years)	Sex distribution (males; n%)	Disease duration	Baseline severity/score
Březina et al. (2021) [23]	FMT	23	39 (median, range: 25-63)	12 (52%) male	9 years (median, range: 1-20)	6 (median, range: 4-10)
	5-ASA enema	22	39.5 (median, range: 27-70)	11 (50%) male	4.5 years (median, range: 0.6-20)	6 (median, range: 4-10)
Crothers et al. (2021) [22]	Active (FMT)	6	41 (±15)	4 (67%) male	8.9 (±9.1) years	6.3 (±2.0)
	Placebo	6	52 (±15)	3 (50%) male	9.8 (±10.6) years	6.7 (±1.2)
Haifer et al. (2022) [21]	FMT	15	37.1 (median, IQR: 31.8-46.8)	9 (60%) male	5.0 years (median, IQR: 3.0-12.0)	5 (median, IQR: 5-9)
	Placebo	20	36.7 (median, IQR: 25.1-42.0)	9 (45%) male	4.0 years (median, IQR: 2.0-9.8)	7 (median, IQR: 5-8)
Kedia et al. (2022) [19]	FMT-AID	35	33.9 (±11.3)	20 (57.1%) male	48 months (median, IQR: 24-96)	SCCAI: 6 (median)
	Optimized SMT	31	37.8 (±10.7)	20 (64.5%) male	36 months (median, IQR: 30-84)	SCCAI: 6 (median)
Moayyedi et al. (2015) [18]	FMT	38	42.2 (±15.0)	18 (47%) male	7.9 (±5.6) years	8.24 (±2.61)
	Placebo	37	35.8 (±12.1)	26 (70%) male	7.0 (±6.8) years	7.86 (±2.28)
Fang et al. (2021) [25]	FMT monotherapy	10	51.5 (±12.7)	2 (20%) male	5.9 (±7.3) years	9.5 (±2.5)
	Control group	10	44.6 (±14.9)	2 (20%) male	6.9 (±9.3) years	8.6 (±7.0)
Sarbagili Shabat et al. (2022) [20]	Group 1: standard FT	17	43.5 (±10.5)	12 (70.6%) male	7.1 years (median, IQR: 3.3-18.0)	SCCAI: 7 (median)
	Group 2: diet + FT	19	33.3 (±9.8)	14 (73.7%) male	11.2 years (median, IQR: 4.7-16.9)	SCCAI: 8 (median)
	Group 3: diet alone	15	40.4 (±12.5)	11 (73.3%) male	7.9 years (median, IQR: 1.9-9.4)	SCCAI: 6 (median)
Sood et al. (2019) [27]	Maintenance FMT	31	33.0 (±12.4)	22 (70.9%) male	4.1 (±2.9) years	1.9 (±0.3) (in remission)
	Placebo	30	34.6 (±12.3)	22 (73.3%) male	4.5 (±3.5) years	1.8 (±0.4) (in remission)
Lahtinen et al. (2023) [11]	FMT group	24	43.0 (±12.9)	14 (58.3%)	39.2 (±51.0) months	Clinical Mayo score: <3 for 17 patients; ≥3 for 7 patients; IBDQ score: 169.4 (±28.8)
	Placebo group (autologous)	24	43.1 (±12.1)	12 (50.0%)	114.0 (±117.6) months	Clinical Mayo score: <3 for 16 patients; ≥3 for 8 patients; IBDQ score: 162.7 (±39.8)

Primary endpoints

Clinical Remission

**Figure 2:**
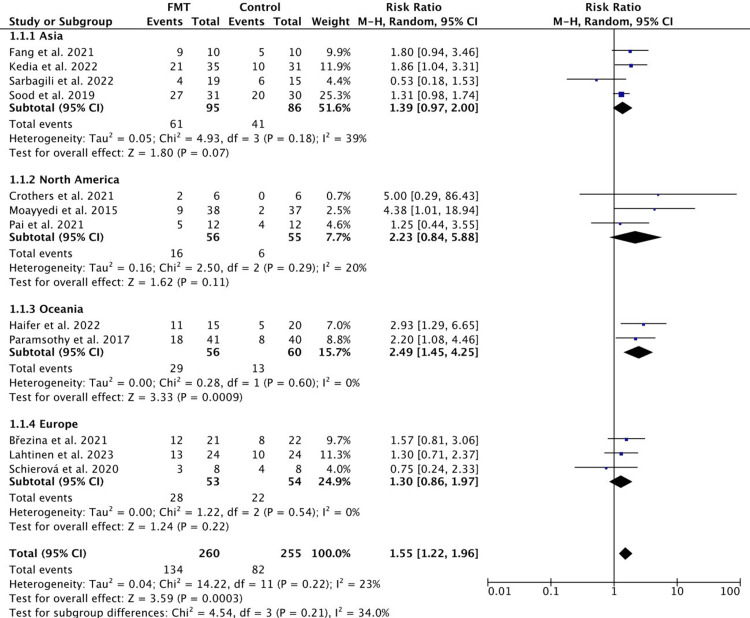
Clinical Remission-Overall and Geographical Variations References [11,17-25,27] CI: confidence interval; FMT: fecal microbiota transplantation

**Figure 3:**
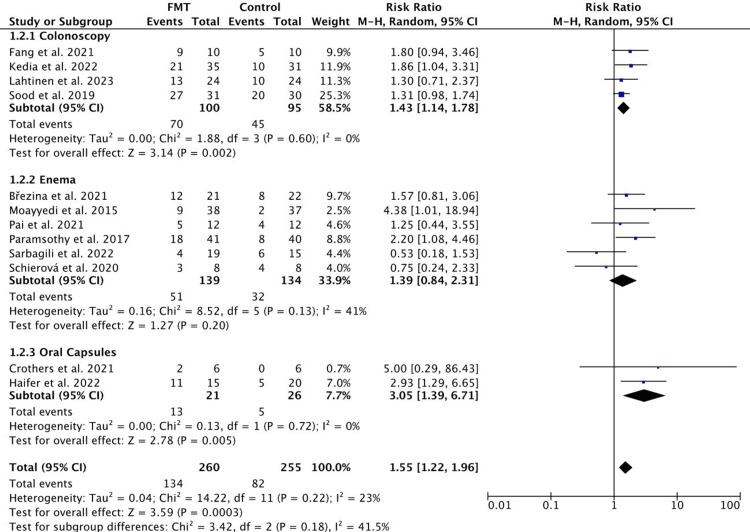
Subgroup Analysis of Clinical Remission-Donor Type References [11,17-27] CI: confidence interval; FMT: fecal microbiota transplantation

A total of 467 patients were included in 11 [17-27] RCTs (236 in the FMT group and 231 in the placebo group). Most studies reported the geographical distribution as being from Asia (4), then North America (3), Oceania (2), and Europe (2). Regarding the route of FMT delivery, six studies delivered FMT via colonoscopy, four via enema, and the rest via oral capsules. Overall, the placebo group compared to the FMT group showed clinically significant remission (RR 1.55; 95% confidence interval (CI) 1.22-1.96; p = 0.0003). In the Oceania region, significant clinical remission was reported to be the highest in RCTs (RR 2.49; 1.45-4.25; p = 0.0009) (Figure 2). In the analysis based upon the type of donor of the FMT, both single (RR 1.45; 95% CI 1.06-1.98; p = 0.02) and multi-donor (RR 1.89; 95% CI 1.26-2.84; p = 0.002) FMT showed significant clinical remission. However, multi-donor FMT was associated with greater clinical remission (RR 1.89 vs. 1.45) compared to single-donor FMT (Figure 3).

**Figure 4:**
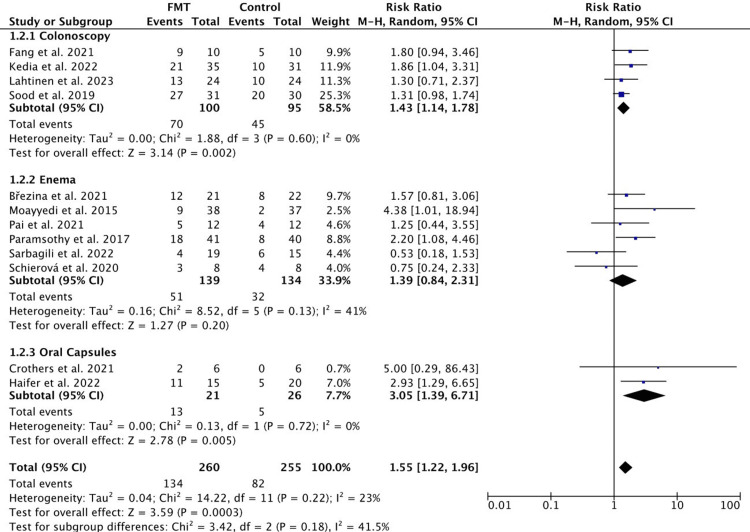
Subgroup Analysis of Clinical Remission-Route of Administration References [11,17-27] CI: confidence interval; FMT: fecal microbiota transplantation

**Figure 5:**
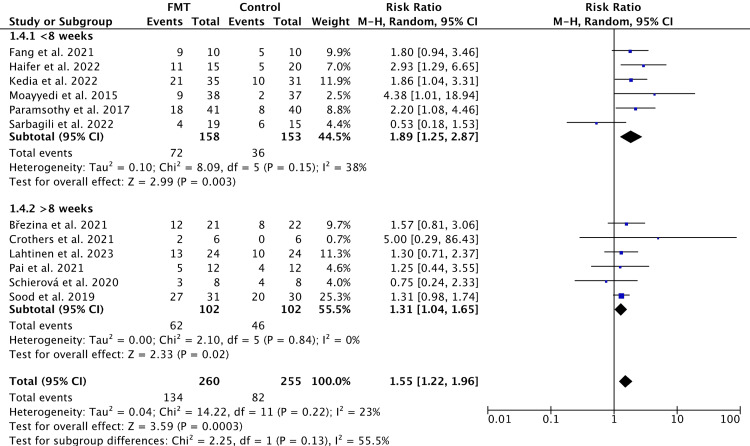
Subgroup Analysis of Clinical Remission-Follow-Up References [11,17-27] FMT: fecal microbiota transplantation; CI: confidence interval

In terms of the route of administration, FMT delivered through oral capsules showed the highest rate of remission (RR 3.05; 95% CI 1.38-6.71; p = 0.005), and subsequently, colonoscopy was rated with an RR 1.43 (95% CI 1.14-1.78; p = 0.002) (Figure 4). With respect to the duration of follow-up, FMT was the only variable to display significant clinical remission in studies with both less than eight weeks of follow-up (RR 1.89; 95% CI 1.25-2.87; p = 0.003) and more than eight weeks of follow-up (RR 1.31; 95% CI 1.04-1.65; p = 0.02) (Figure 5).

Endoscopic Remission

**Figure 6:**
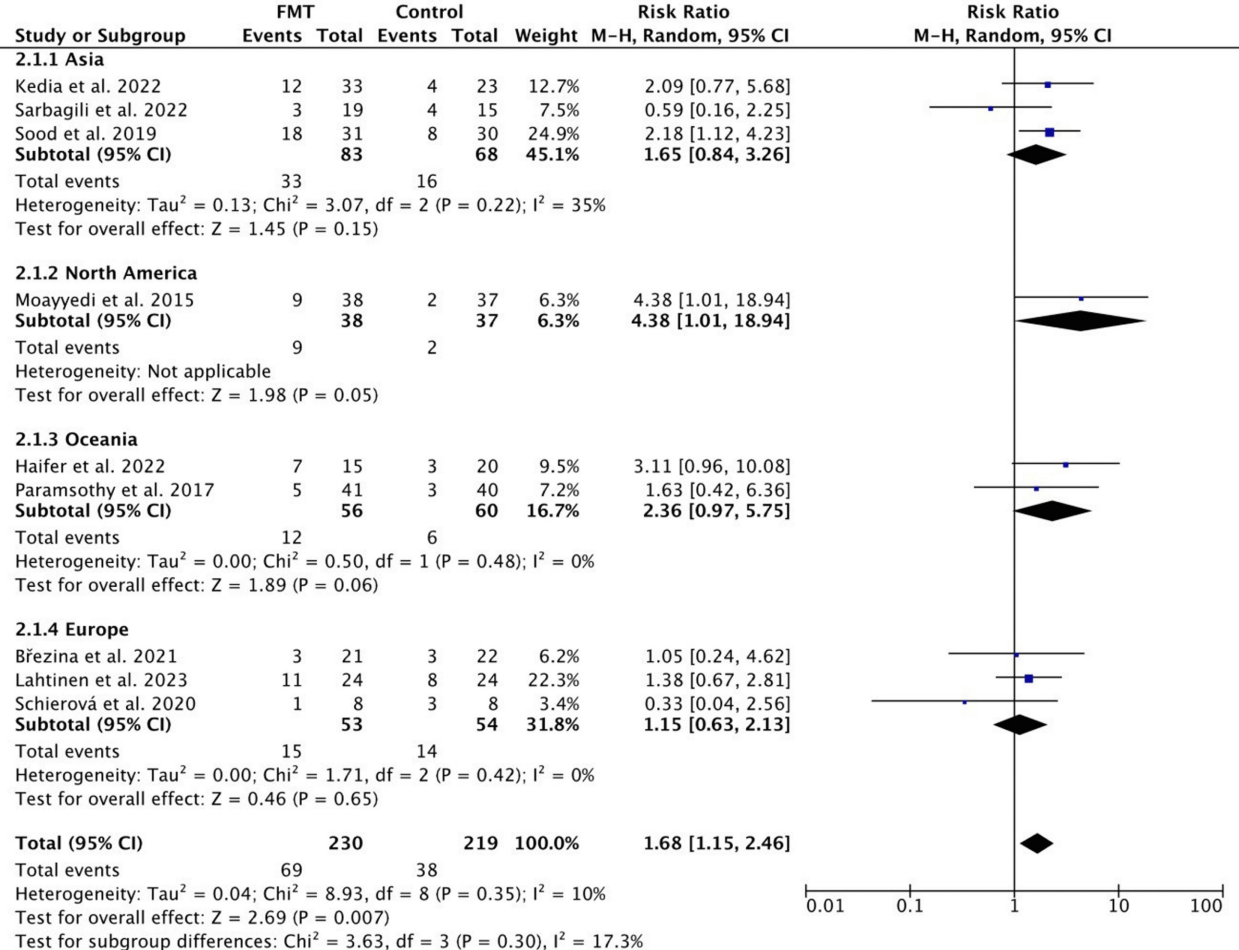
Subgroup Analysis of Endoscopic Remission-Overall References [11,17-21,23,26,27] CI: confidence interval; FMT: fecal microbiota transplantation

**Figure 7:**
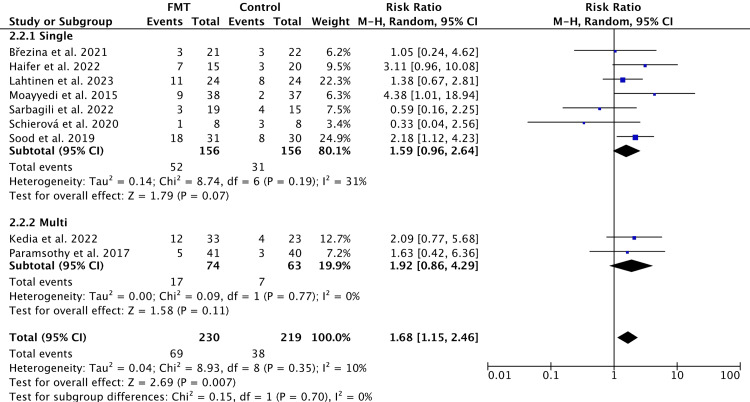
Subgroup Analysis of Endoscopic Remission-Donor Type References [11,17-21,23,26,27] FMT: fecal microbiota transplantation; CI: confidence interval

**Figure 8:**
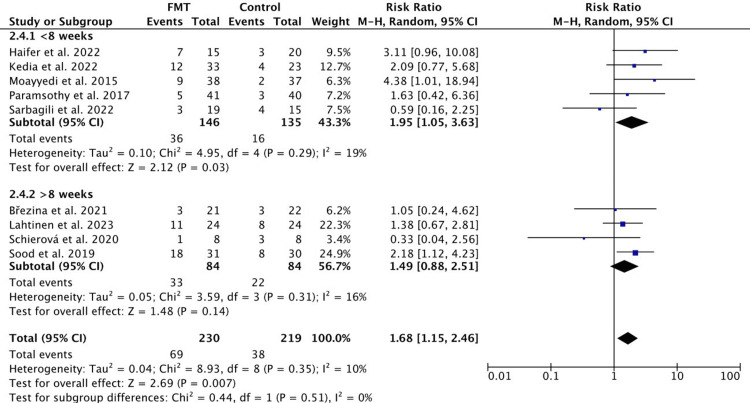
Subgroup Analysis of Endoscopic Remission-Follow-Up References [11,17-21,23,26,27] CI: confidence interval; FMT: fecal microbiota transplantation

**Figure 9:**
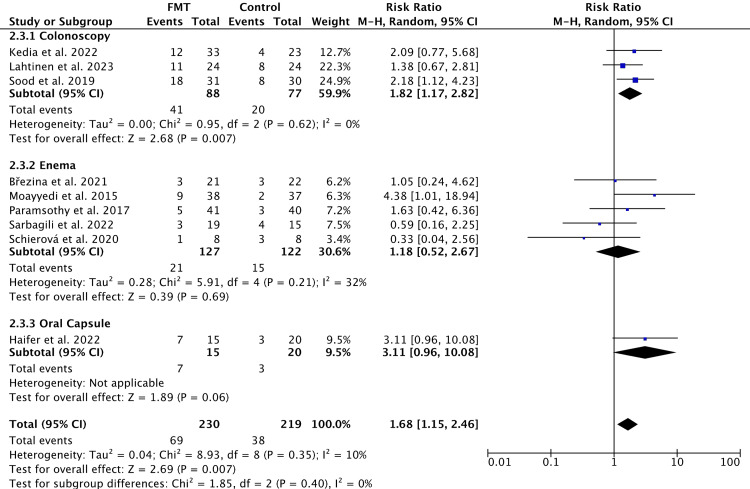
Subgroup Analysis of Endoscopic Remission-Route of Administration References [11,17-21,23,26,27] FMT: fecal microbiota transplantation; CI: confidence interval

The FMT group showed considerable endoscopic remission over the placebo (RR 1.68; 95% CI 1.15-2.46; p = 0.007). Endoscopic remission due to placebo treatment varied by region. The highest remission among North American RCTs was reported (RR 4.38; 95% CI 1.01-18.94; p = 0.05) (Figure 6). In most North American RCTs, the FMT group had multi-donor FMT (RR 1.92; 95% CI 0.86-4.29; p = 0.11), and single-donor FMT showed RR 1.59; 95% CI 0.96-2.64; p = 0.07 (Figure 7). FMT showed endoscopic remission after eight weeks in one study (RR 1.95; 95% CI 1.05-3.63; p = 0.03) (Figure 8). FMT via colonoscopy showed significant endoscopic remission (RR 1.82; 95% CI 1.17-2.82; p = 0.007) (Figure 9).

Secondary endpoints

**Figure 10:**
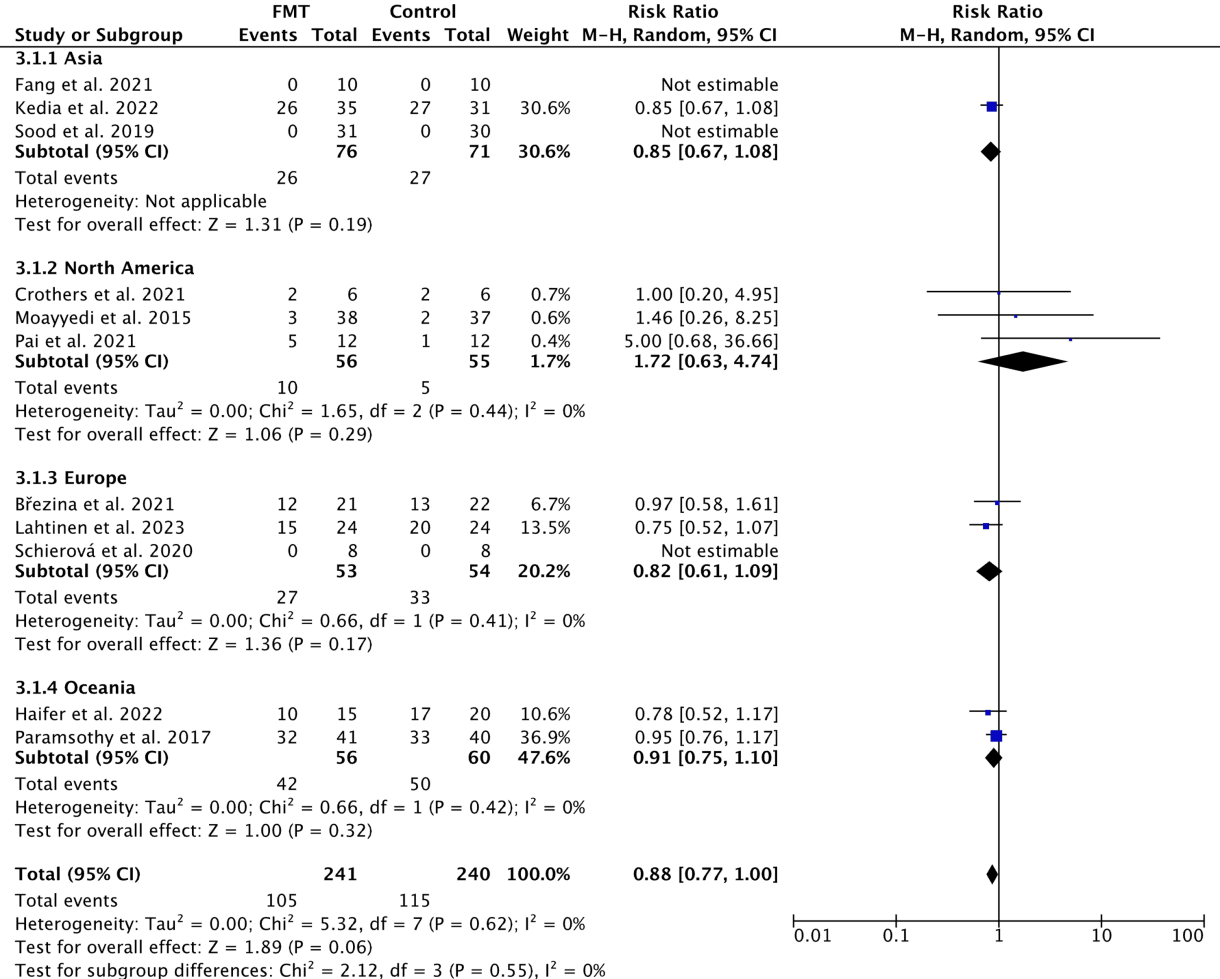
Overall Adverse Events References [11,17-19,21-27] FMT: fecal microbiota transplantation; CI: confidence interval

**Figure 11:**
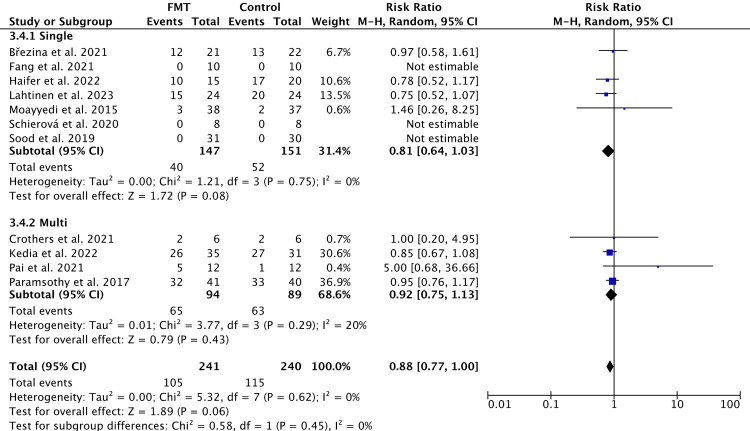
Subgroup Analysis of Adverse Events-Donor Type References [11,17-19,21-27] FMT: fecal microbiota transplantation; CI: confidence interval

**Figure 12:**
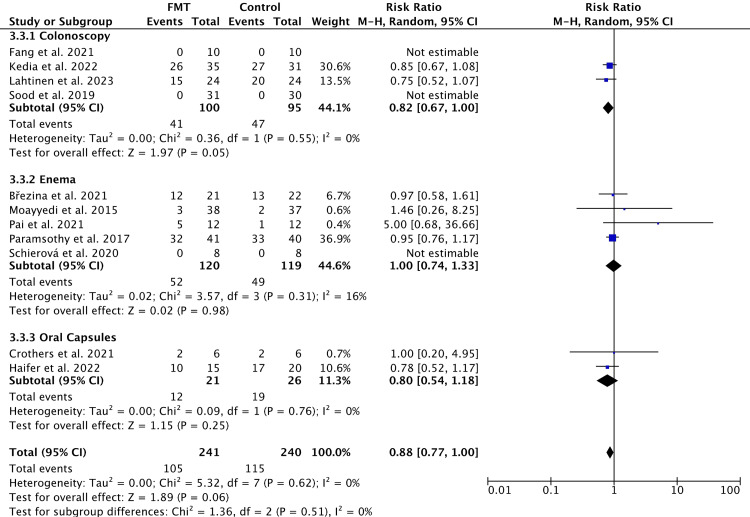
Subgroup Analysis of Adverse Events-Route of Administration References [11,17-19,21-27] FMT: fecal microbiota transplantation; CI: confidence interval

**Figure 13:**
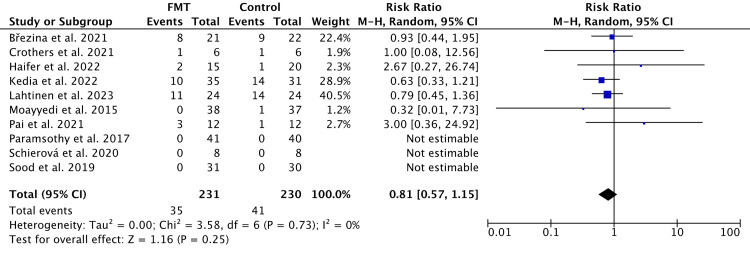
Adverse Events-Worsening Ulcerative Colitis References [11,17-19,21-24,26,27] FMT: fecal microbiota transplantation; CI: confidence interval

In general, the rate of AEs was similar between the FMT and control groups. The overall AE pooled RR was 0.88 (95% CI 0.77-1.00; p = 0.06), which means FMT does not lead to a statistically significant increase in AEs. When analyzing by donor type, single-donor FMT (RR 0.81; 95% CI 0.64-1.03; p = 0.08) had a marginally better pooled RR for AEs than multi-donor FMT (RR 0.92; 95% CI 0.75-1.13; p = 0.43). In terms of administration route and AEs, oral capsules (RR 0.80; 95% CI 0.54-1.18; p = 0.09) had the lowest pooled RR, and then colonoscopy (RR 0.82; 95% CI 0.67-1.00; p = 0.55). For FMT, the pooled RR for worsening UC was 0.81 (95% CI 0.57-1.15; p = 0.73), and therefore, UC does not statistically significantly worsen compared to control (Figures 10-13).

Heterogeneity and RoB assessment

**Figure 14:**
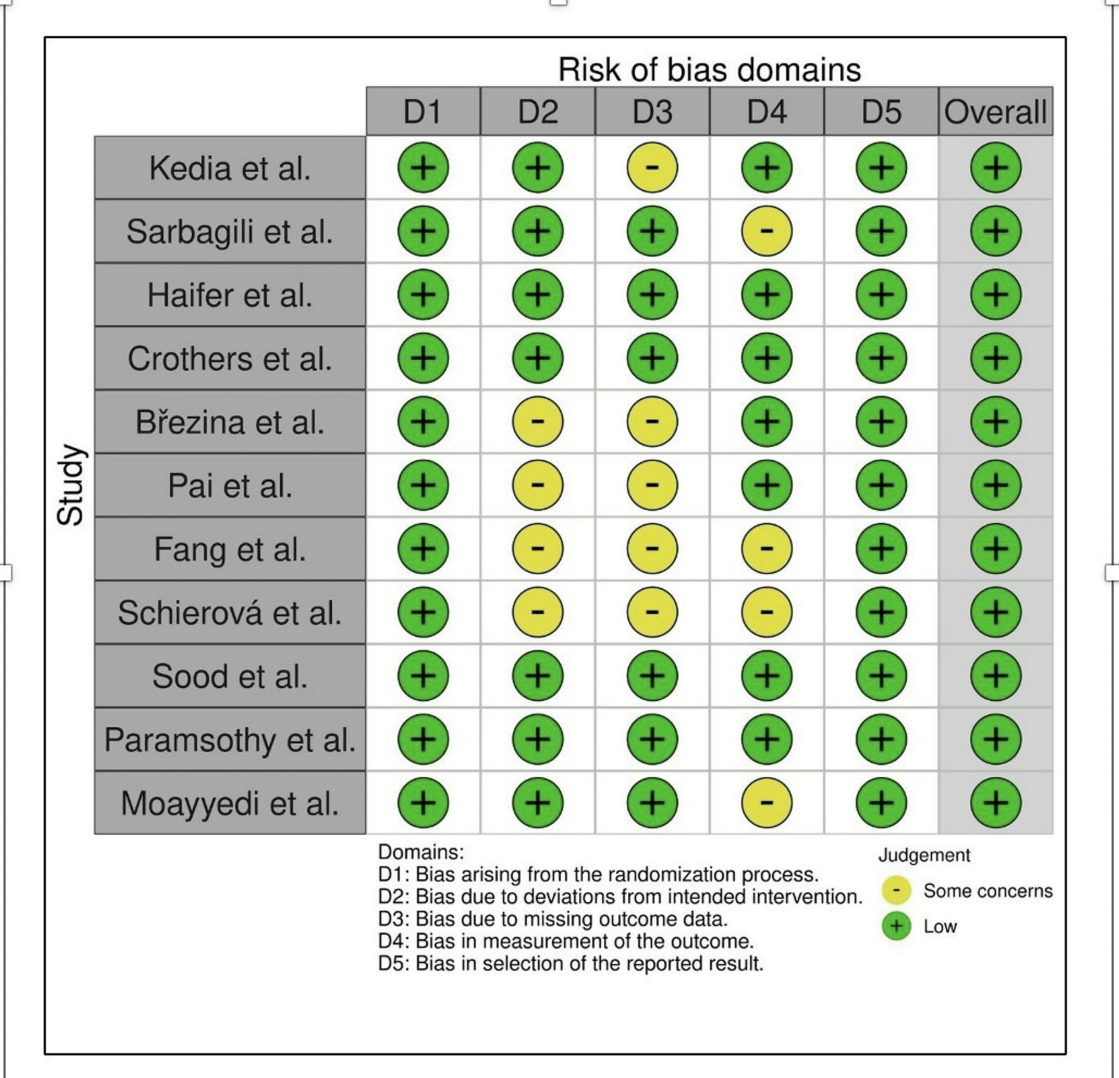
RoB Assessment References [17-27] RoB: risk of bias

The heterogeneity of outcomes and subgroups ranged from no heterogeneity (I^2^ = 0%) to moderate heterogeneity (I^2^ up to 41%). Table 5 provides specific I^2^ and p-values for each analysis. In terms of RoB evaluation, all studies were assessed to have a low overall RoB (Figure 14).

**Table 5:** Heterogeneity Assessment UC: ulcerative colitis

Outcome & subgrouping	I^2^ (%)	p-value
Clinical remission	23	0.0003
By geographic region		
Asia	39	0.18
North America	20	0.29
Oceania	0	0.6
Europe	0	0.54
By donor type		
Single donor	37	0.14
Multi-donor	0	0.99
By follow-up duration		
<8 weeks	38	0.15
>8 weeks	0	0.84
By route of administration		
Colonoscopy	0	0.6
Enema	41	0.16
Oral capsules	0	0.72
Endoscopic remission	10	0.007
By geographic region		
Asia	35	0.22
North America	N/A	N/A
Oceania	0	0.48
Europe	0	0.42
By donor type		
Single donor	31	0.19
Multi-donor	0	0.77
By follow-up duration		
<8 weeks	19	0.29
>8 weeks	16	0.31
By route of administration		
Colonoscopy	0	0.95
Enema	32	0.21
Oral capsules	N/A	N/A
Adverse events	0	0.06
By donor type		
Single donor	0	0.08
Multi-donor	20	0.43
By route of administration		
Colonoscopy	0	0.55
Enema	16	0.2
Oral capsules	0	0.09
Worsening UC	0	0.73

Discussion

The purpose of this meta-analysis was to evaluate the efficacy and the safety of FMT across studies to establish clinical and endoscopic remission of patients suffering from UC and to investigate variables that affect the outcomes of FMT. Supporting the safety and the therapeutic impact of FMT on UC, we describe the effective impact of various FMT administration parameters. Significant clinical and endoscopic remission results were observed with FMT in comparison to placebo. However, earlier RCTs have reported no significant difference noted [28]. However, recent meta-analyses [9,10,29,30] observed comparable results. Nonetheless, the subgroup analysis provided new and insightful observations.

The variation in the RRs could be due to the geographical region, variability in the donor screening process, distinct characteristics of the recipients, or microbiome environmental changes. Additionally, in both single and multiple donors, FMT showed measurable clinical remission outcomes. In this regard, multiple donors provided clinically better results, owing to increased microbial diversity from the donors, the presence of more beneficial microbes, or the expansion of the transplanted microbial community. Furthermore, the administration route of the FMT was established as an important parameter.

The improved efficiency of oral capsules is considered a positive finding because this method is better than a colonoscopy or enema, as it is less invasive and facilitates adherence and accessibility to treatment options. However, concerning endoscopic remission, the trend of higher endoscopic remission is observed in these studies with shorter follow-up (<8 weeks) than in studies with longer follow-up (>8 weeks), which indicates that significant initial endoscopic improvement may be achieved, but sustaining improvement from endoscopic changes at longitudinal intervals may be areas that are less studied and may require additional optimization of the FMT protocols such as more frequent administrations of FMT. As for safety, the meta-analysis demonstrated that the occurrence of AEs was approximately the same in both FMT and control groups, allowing us to consider that FMT is a relatively safe approach to UC patients and gives us a reason to consider FMT in the clinical setting.

The authors of the meta-analysis have commissioned other researchers to consider addressing other party limitations. While most of the limitations of a meta-analysis have been discussed, it is reasonable to propose other limitations that have not been studied. For instance, an observed moderate heterogeneity in clinical remission (I^2^ = 23%) suggests variability in reporting clinical remission in other participants, which could be due to differences in population, the severity of the disease, the selection of the donor population, FMT, the selection and preparation of the different types of FMT, and different concomitant medications used during the therapy process. While multiple subgroup analyses were used to eliminate or minimize the sources of heterogeneity, the presence of other unmeasured factors that could explain differences in the treatment of patients who did not experience improvement may exist.

Second, a small quantity of included RCTs, especially in the noted subgroups (endoscopic remission and oral capsules in North America), may restrict the universality and statistical power of specific results. Third, varying duration of follow-up across studies may have a possible influence on the long-term evaluation of remission and the occurrence of adverse effects. For instance, some studies have follow-up periods of under eight weeks, which may not allow for the capture of prolonged remission or adverse effects that arise later. Fourth, the specific active elements of the donor microbiome that confer the therapeutic effects of FMT remain predominantly unknown. This lack of understanding of the mechanisms involved makes the precise tailoring of donor selection and microbial composition to specific patient needs challenging. Fifth, the lack of a uniform standard for FMT administration across studies regarding donor selection, microbial preparation, administration, dosage, and frequency introduces variability that may impact the consistency and comparability of findings.

Several important aspects of the clinical use of FMT in the treatment of UC remain to be investigated in future studies based on the data of the present meta-analysis. First, there is a need for larger multi-center RCTs to evaluate the effectiveness and the safety of FMT in different patient populations and to develop suitable treatment protocols. To enhance comparability and reduce heterogeneity in clinical trials, donor screening, stool preparation, and administration should be standardized. Second, in-depth studies are needed to gain insights into the specific microorganisms, metabolites, and functional mechanisms within the microbial community that are involved in the modulation process for UC. Such studies may include the use of advanced metagenomic, meta-transcriptomic, and metabolomic approaches to identify “super donors” and/or design targeted microbial consortia. Third, developing a strategy for administering FMT via optimizing the delivery mechanism is of utmost importance. In this context, oral capsules show promising outcomes and are likely to enhance patient (dis)comfort. Clinical studies on the differing effectiveness of other routes of delivery, differing doses, and delivery frequency are warranted. Fourth, studies concerning the long-standing effects of FMT on UC and the rare potential adverse effects accompanying the treatment should be conducted to fully comprehend the effectiveness and safety of FMT. Lastly, the invention of FMT response biomarkers will allow for an individualized approach in FMT treatment of UC. As such, it will increase the probability of success and assist in the proper patient selection.

## Conclusions

In summary, the advancement of FMT in UC requires a focus on process consistency in research studies. Primary objectives should be on the establishment of standard operating procedures, donor selection, and the packaging and administration of FMT via oral capsules. Alongside this, a greater foundational understanding of the therapeutic microbes through advanced “omics” studies is crucial for developing predictive biomarkers and identifying key microbial components. Ultimately, this research will improve the safety and efficacy of FMT for UC.
